# Cytotoxic Sesterterpenoids from a Sponge *Hippospongia* sp*.*

**DOI:** 10.3390/md10050987

**Published:** 2012-04-27

**Authors:** Yu-Chia Chang, Shang-Wei Tseng, Li-Lian Liu, Yalan Chou, Yuan-Shing Ho, Mei-Chin Lu, Jui-Hsin Su

**Affiliations:** 1 National Museum of Marine Biology & Aquarium, Pingtung 944, Taiwan; Email: jay0404@gmail.com (Y.-C.C.); fallboys2006@hotmail.com (S.-W.T.); jinx6609@nmmba.gov.tw (M.-C.L.); 2 Doctoral Degree Program in Marine Biotechnology, National Sun Yat-sen University and Academia Sinica, Kaohsiung 804, Taiwan; 3 Graduate Institute of Marine Biotechnology, National Dong Hwa University, Pingtung 944, Taiwan; 4 Institute of Marine Biology, National Sun Yat-sen University, Kaohsiung 804, Taiwan; Email: lilian@mail.nsysu.edu.tw (L.-L.L.); ylchou@gmail.com (Y.C.); 5 Asia-Pacific Ocean Research Center, National Sun Yat-sen University, Kaohsiung 804, Taiwan; 6 Eastern Marine Biology Research Center, Fisheries Research Institute, Taitung 961, Taiwan; Email: yuanho18@gmail.com

**Keywords:** sesterterpenoid, scalarane, sponge, *Hippospongia*

## Abstract

One new pentacyclic sesterterpene, hippospongide A (**1**), and one new scalarane sesterterpenoid, hippospongide B (**2**), along with six previously reported known scalarane–type sesterterpenes (**3**–**8**), were isolated from a sponge *Hippospongia* sp*.* The structures of these compounds were elucidated on the basis of their spectroscopic data and comparison of the NMR data with those of known analogues. These metabolites are the first pentacyclic sesterterpene and scalarane-type sesterterpenes to be reported from this genus. Compounds **3**–**5** exhibited significant cytotoxicity against DLD-1, HCT-116, T-47D and K562 cancer cell lines.

## 1. Introduction

**Figure 1:**
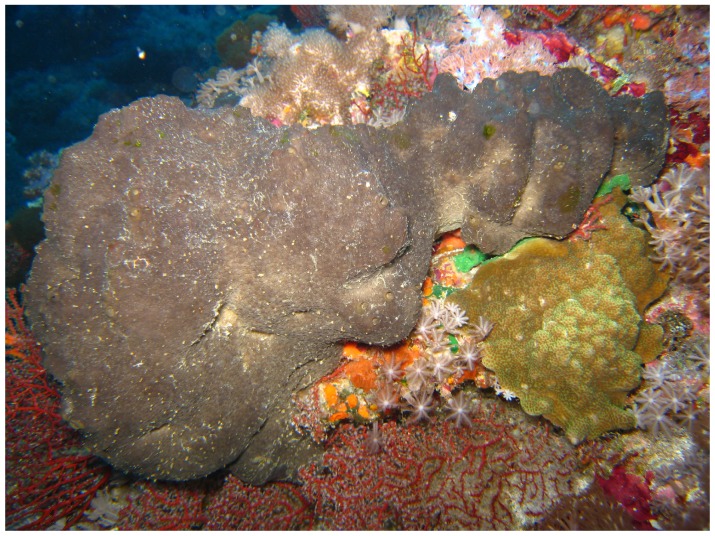
Sponge *Hippospongia* sp*.*

In previous reports, scalarane sesterterpenoids have been identified from sponges and nudibranchs [1]. Research into the pharmacological properties of this class of natural products is of particular interest. In fact, many scalarane metabolites show a variety of biological activities, such as antimicrobial, cytotoxic, antifeedant, ichthyotoxic, anti-inflammatory, antitubercular, platelet aggregation inhibition, RCE-protease inhibition and nerve growth factor synthesis-stimulating [1]. Our investigation of the chemical constituents of a sponge *Hippospongia* sp*.* (Figure 1) yielded one new pentacyclic sesterterpene, hippospongide A (**1**), and one new scalarane sesterterpenoid, hiposppongide B (**2**), along with six known sesterterpenoids, heteronemin (**3**) [2], heteronemin acetate (**4**) [3], hyrtiosin E (**5**) [4], 12-deacetoxyscalarin 19-acetate (**6**) [5], hyrtiosal (**7**) [6] and scalarafuran (**8**) [7]. The cytotoxicity of metabolites **1**–**8** against human colon adenocarcinoma (DLD-1 and HCT-116), hormone-dependent breast cancer (T-47D) and human chronic myelogenous leukemia (K562) cell lines was evaluated.

## 2. Results and Discussion

**Chart 1:**
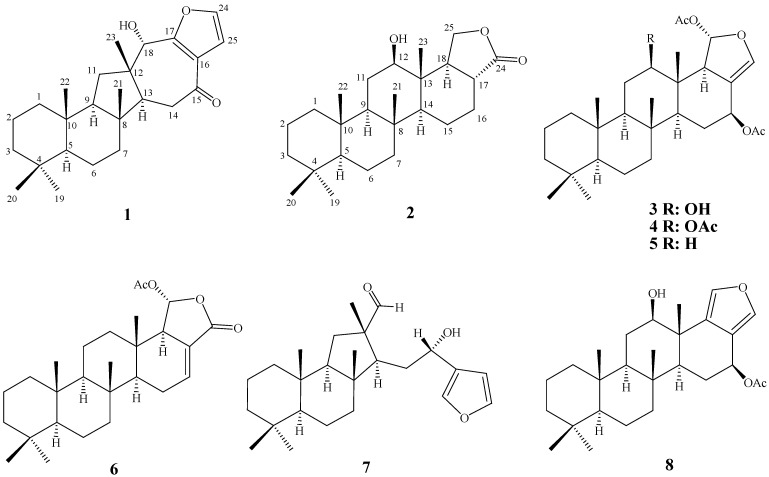
Structures of metabolites **1**–**8**.

The EtOAc extract of the freeze-dried specimen was fractionated by silica gel column chromatography and the eluted fractions were further separated utilizing normal phase HPLC to yield metabolites **1**–**8** (Chart 1). 

**Figure 2:**
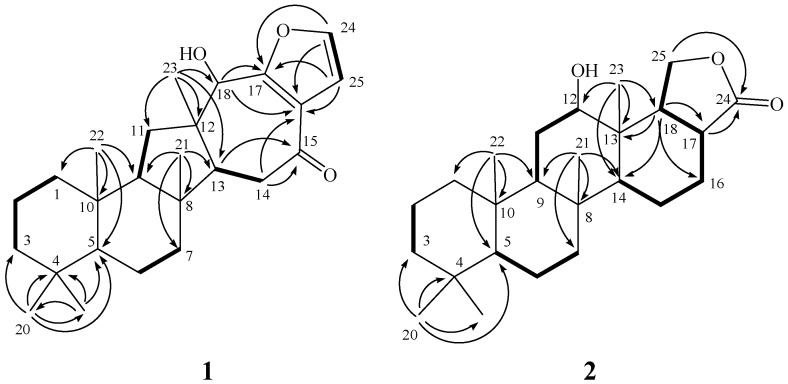
Selected ^1^H−^1^H COSY (▬) and HMBC (→) correlations of **1** and **2**.

The new metabolite hippospongide A (**1**) had a molecular formula of C_2__5_H_3__6_O_3_ as determined by HRESIMS and NMR spectroscopic data. The IR spectrum of **1** showed absorption bands at 3386 cm^−1^, suggesting the presence of a hydroxy group. The ^13^C NMR data of **1** showed the presence of 25 carbons (Table 1): five methyls, seven sp^3^ methylenes, four sp^3^ methines (including one oxygenated carbon at δ 75.9), two sp^2^ methines, and four sp^3^ quaternary carbons. The remaining three signals appearing in the downfield region of the spectrum are due to the quaternary carbons of two olefinic carbons (δ 122.9 and 159.0) and one ketone carbonyl (δ 196.8). From the ^1^H NMR (Table 1) spectrum of **1**, the ^1^H NMR data revealed the presence of two olefinic methine protons (δ 7.33 Hz; d, *J* = 1.5 Hz; 6.76 Hz; d, *J* = 1.5 Hz). Furthermore, one oxygenated methine (δ 4.58, s) was also designated from the ^1^H NMR signal. Careful analysis of the ^1^H–^1^H COSY correlations observed for **1** led to the establishment of five partial structures, as shown in Figure 2. The molecular framework of **1** was further established by a HMBC experiment (Figure 2). The five rings and their connectivities were elucidated on the basis of the following key HMBC correlations: both methyls H_3_-19 and H_3_-20 to C-3, C-4 and C-5, H_3_-21 to C-7, C-8, C-9 and C-13, H_3_-22 to C-1, C-5, C-9 and C-10, H_3_-23 to C-11, C-12, C-13 and C-18, H-13 to C-15, H-14 to C-15 and C-16, H-18 to C-17 and C-16, and both olefinic methines H-24 and H-25 to C-16 and C-17. Thus, **1** was found to possess two double bonds at C-16/C-17 and C-24/C-25, one hydroxy group at C-18, and one ketone group at C-15. Linking all the above functional groups to the sesterterpene skeleton thus yielded the gross structure of **1**.

**Figure 3:**
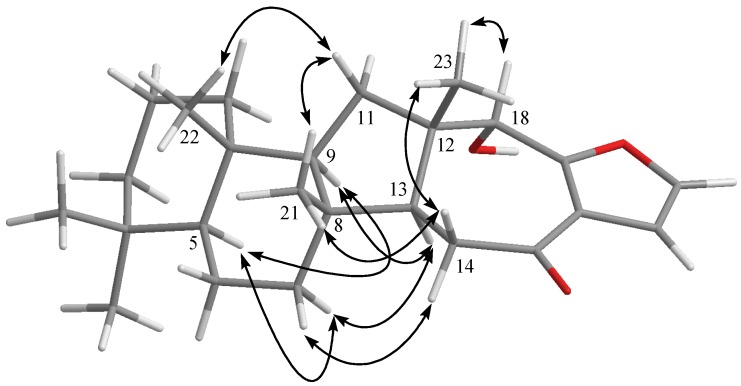
Computer-generated model of **1** using MM2 force field calculations and key NOE correlations.

The relative configuration of **1**, elucidated mainly from the NOESY spectrum, was corroborated by MM2 force field calculations, which suggested the most stable conformation to be that shown in Figure 2. In the NOESY spectrum, H-9 showed NOEs with H-5 and H-13 but not with three methyls H_3_-21, H_3_-22 and H_3_-23. Thus, assuming an α-orientation of H-5, both H-9 and H-13 must also be on the α face whilst the three methyls H_3_-21, H_3_-22 and H_3_-23 must be located on the β face. Moreover, the NOE correlations of H_3_-23 with H-18 indicated the β-orientation of H-18. On the basis of the above findings and other detailed NOE correlations (Figure 3), the relative structure of **1** was determined. After determining the structure of **1**, we discovered that its molecular framework has been obtained as known sesterterpenoids salmahyrtisol A and similan A, which were isolated previously from sponges *Hyrtios erecta* [8] and *Hyrtios gumminae* [9], respectively*.*

**Table 1:** ^1^H and ^13^C NMR data for **1** and **2**.

Position	1		2	
	δ_H_ (*J* in Hz) ^a^	δ_C_ (mult.) ^b^	δ_H_ (*J* in Hz) ^a^	δ_C_ (mult.) ^b^
1	1.46 m; 0.98 m	40.2 (CH_2_) ^c^	1.65 m	39.9 (CH_2_)
2	1.65 m; 1.40 m	18.4 (CH_2_)	1.54 m; 1.38 m	18.2 (CH_2_)
3	1.38 m; 1.19 m	42.5 (CH_2_)	1.36 m; 1.12 m	42.0 (CH_2_)
4		33.1 (C)		33.3 (C)
5	0.92 m	57.6 (CH)	0.80 m	56.5 (CH)
6	1.57 m; 1.38 m	18.8 (CH_2_)	1.61 m; 1.42 m	18.6 (CH_2_)
7	1.68 m; 1.10 m	40.1 (CH_2_)	1.74 m; 0.90 m	41.7 (CH_2_)
8		44.8 (C)		37.3 (C)
9	1.45 m	61.0 (CH)	0.88 m	58.9 (CH)
10		36.8 (C)		37.5 (C)
11	1.99 d (6.0); 1.43 m	35.0 (CH_2_)	1.70 m; 1.45 m	27.5 (CH_2_)
12		43.0 (C)	3.40 br d (10.5)	80.5 (CH)
13	2.20 dd (13.0, 2.5)	47.5 (CH)		42.0 (C)
14	2.64 dd (13.5, 13.0)	39.6 (CH_2_)	0.80 m	58.1(CH)
	2.54 dd (13.5, 2.5)			
15		196.8 (C)	1.78 m; 1.36 m	20.0 (CH_2_)
16		122.9 (C)	2.20 m; 1.22 m	25.6 (CH_2_)
17		159.0 (C)	2.22 m	39.2 (CH)
18	4.58 s	75.9 (CH)	1.86 m	55.3 (CH)
19	0.85 s	33.5 (CH_3_)	0.84 s	33.2 (CH_3_)
20	0.84 s	21.3 (CH_3_)	0.80 s	21.3 (CH_3_)
21	0.85 s	16.2 (CH_3_)	0.84 s	17.3 (CH_3_)
22	0.87 s	15.6 (CH_3_)	0.84 s	16.3 (CH_3_)
23	1.14 s	23.4 (CH_3_)	0.91 s	9.8 (CH_3_)
24	7.33 d (1.5)	142.3 (CH)		177.8 (C)
25	6.76 d (1.5)	110.9 (CH)	4.38 dd (9.5, 7.0)	70.0 (CH_2_)
			4.09 dd (11.0, 10.0)	

*^a^* 500 MHz in CDCl_3_; *^b^* 125 MHz in CDCl_3_; *^c^* Numbers of attached protons were deduced by DEPT experiments.

**Figure 4:**
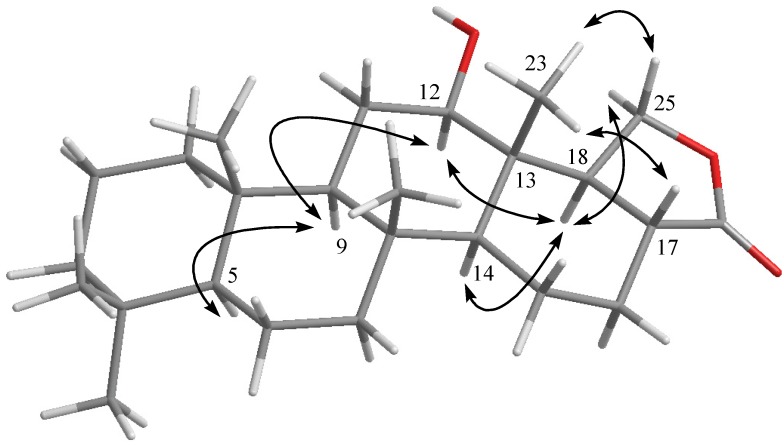
Key NOE correlations of **2**.

Hippospongide B (**2**) was isolated as a white powder with the molecular formula C_2__5_H_40_O_3_, which possesses six degrees of unsaturation, as indicated by HRESIMS (*m*/*z* 411.2878, [M + Na]^+^) and NMR spectroscopic data (Table 1). Moreover, it was found that the NMR data of the tricyclic skeleton (C-1 to C-14) of **2** were quite similar to those of **3** and **8**, indicating the same substitution and stereochemistry at C-5, C-8, C-9, C-10, C-12, C-13 and C-14. Furthermore, analysis of the ^1^H–^1^H COSY and HMBC correlations established the remaining structure, including another two rings from C-13 to C-18 (Figure 2). Finally, the relative stereochemistries at C-17 and C-18 were resolved by careful interpretation of the NOE correlations (Figure 4). Key NOE correlations for **2** showed interactions between H-18 to H-12 and H-14. Thus, H-18 should be located on the α face. NOE correlations were also detected between H-17 and H_3_-23, revealing the β-orientation of H-17, as suggested by a molecular model of **2**. After structural determination of **2**, we found that this compound had been obtained previously by hydrogenation of the natural product hydroxylactone IV [10]. In the original report, the authors gave a planar structure. However, our study led to the isolation of **2** for the first time from natural sources. In addition, we successfully elucidated the full structure of **2**. Moreover, our work also provides full assignment for the ^1^H and ^13^C NMR spectral data of **2**.

The cytotoxicities of compounds **1**–**8** against DLD-1, HCT-116, T-47D and K562 cancer cells are shown in Table 2. The results showed that compounds **3**–**5** were found to exhibit cytotoxicity against all or part of the above carcinoma cell lines, while compound **3** (IC_50_ values 0.001, 0.001, 0.001 and 0.001 μM against the above carcinoma cell lines, respectively) was the most potent.

**Table 2:** Cytotoxicity (IC_50_ μM) of compounds **1**–**5**.

Compound	Cell Lines			
	DLD-1	HCT-116	T-47D	K562
**1**	– ^a^	– ^a^	– ^a^	– ^a^
**2**	– ^a^	– ^a^	– ^a^	– ^a^
**3**	0.001	0.001	0.001	0.001
**4**	2.4	2.7	0.3	0.05
**5**	1.1	8.0	0.7	0.7
**6**	– ^a^	– ^a^	– ^a^	– ^a^
**7**	– ^a^	– ^a^	– ^a^	– ^a^
**8**	– ^a^	– ^a^	– ^a^	– ^a^
Actinomycin D	1.9	0.2	0.6	0.03

^a^ IC_50_ > 10 μM.

## 3. Experimental Section

### 3.1. General Experimental Procedures

Optical rotation values were measured with a Jasco P-1010 digital polarimeter. IR spectra were recorded on a Varian Digilab FTS 1000 Fourier transform infrared spectrophotometer. The NMR spectra were recorded on a Varian Unity INOVA 500 FT-NMR instrument at 500 MHz for ^1^H NMR and 125 MHz for ^13^C NMR, respectively, in CDCl_3_. ESIMS data were obtained with a Finnigan LCQ ion-trap mass spectrometer. HRESIMS data were recorded on a LTQ Orbitrap XL mass spectrometer. Gravity column chromatography was performed on silica gel (230–400 mesh, Merck). TLC was carried out on pre-coated Kieselgel 60 F254 (0.2 mm, Merck) and spots were visualized by spraying with 10% H_2_SO_4_ solution followed by heating. High-performance liquid chromatography was performed using a system comprised of a Hitachi L-7100 pump and a Rheodyne 7725 injection port. A preparative normal phase column (250 × 21.2 mm, 5 μm) was used for HPLC.

### 3.2. Animal Material

**Figure 5:**
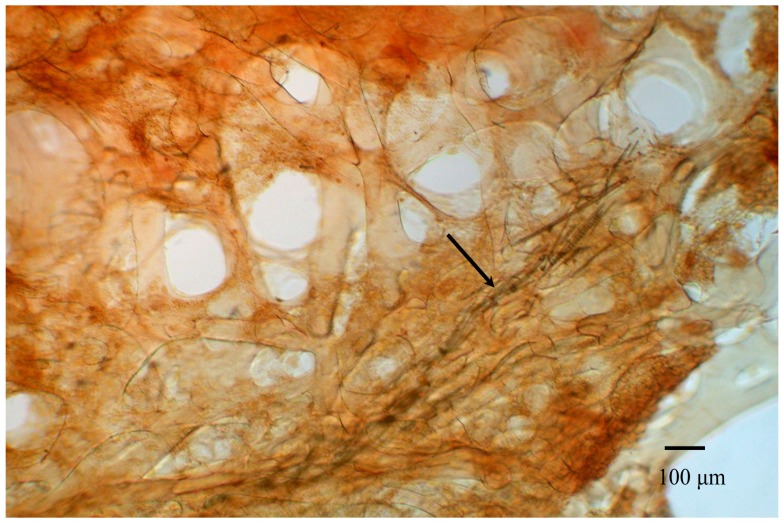
Skeleton architecture of the *Hippospongia* sp. Arrow: foreign broken spicules in primary spongins.

The specimen of *Hippospongia* sp. was collected by scuba diving at a depth of 20 m from coral reefs off the coast of Tai-tung, Taiwan. Voucher specimen was deposited in the National Museum of Marine Biology and Aquarium, Taiwan (specimen No. 2011SP-1). This genus is often confused with *Hyattella* (Lendenfeld, 1888), whereas *Hippospongia* is more elastic and compressible with fewer primary fibers (Figure 5). Taxonomic identification was performed by Li-Lian Liu of the National Sun Yat-sen University, Kaohsiung, Taiwan.

### 3.3. Extraction and Separation

The frozen bodies of *Hippospongia* sp. (1.2 kg fresh wt) were collected and freeze-dried. The freeze-dried material (170 g) was minced and extracted exhaustively with EtOAc (5 × 1 L). The EtOAc extract (15.3 g) was chromatographed over silica gel by column chromatography and eluted with EtOAc in *n*-hexane (0–100%, stepwise), then with acetone in EtOAc (50–100%, stepwise) to yield 13 fractions. Fraction **3** (125.7 mg), eluted with *n*-hexane–EtOAc (10:1), was subjected to normal phase HPLC (*n*-hexane–EtOAc, 7:1) to afford four subfractions (A1–A4). Subfraction A4 (30.5 mg) was separated by normal phase HPLC using *n*-hexane–EtOAc (5:1) to afford **5** (5.9 mg, 0.039% dry wt. of extract) and **6** (2.1 mg, 0.014% dry wt. of extract). Fraction 4 (996 mg), eluting with *n*-hexane–EtOAc (8:1), was further purified by normal phase HPLC (*n*-hexane–EtOAc, 6:1) to afford five subfractions (B1–B5). Subfraction B1 (120 mg) was separated by normal phase HPLC using *n*-hexane–EtOAc (10:1) to afford **1** (1.7 mg, 0.011% dry wt. of extract), **7** (3.0 mg, 0.020% dry wt. of extract) and **8** (20.5 mg, 0.133% dry wt. of extract). Subfraction B2 (20 mg) was also purified by normal phase HPLC using *n*-hexane–EtOAc (7:1) to afford **4** (6.2 mg, 0.041% dry wt. of extract). Fraction 6 (10.5 g), eluting with *n*-hexane–EtOAc (3:1), was further separated by silica gel column chromatography with gradient elution (*n*-hexane–EtOAc, 3:1 to 1:1) to afford **3** (6 g, 39.2% dry wt. of extract). Fraction 8 (524 mg), eluted with *n*-hexane–EtOAc (2:1), was further separated by normal phase HPLC (*n*-hexane–EtOAc, 2:1) to yield six subfractions (C1–C6). Subfraction C3 was separated by normal phase HPLC using *n*-hexane–EtOAc (3:1) to afford **2** (0.8 mg, 0.005% dry wt. of extract). 

Hippospongide A (**1**): white powder; mp 272–274 °C; 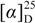 −66 (*c* 0.1, CHCl_3_); IR (neat) ν_max_ 3386, 2922, 2854, 1715, 1642, 1455 and 1385 cm^−^^1^; ^1^H and ^13^C NMR data, see Table 1; ESIMS *m*/*z* 407 (100, [M + Na]^+^); HRESIMS *m*/*z* 407.2560 (calcd for C_2__5_H_3__6_O_3_Na, 407.2562).

Hippospongide B (**2**): white powder; mp 289–291 °C; 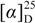
−3 (*c* 0.05, CHCl_3_); IR (neat) ν_max_ 3436, 2927, 1753, 1461 and 1383 cm^−^^1^; ^1^H and ^13^C NMR data, see Table 1; ESIMS *m*/*z* 411 (80, [M + Na]^+^); HRESIMS *m*/*z* 411.2878 (calcd for C_2__5_H_40_O_3_Na, 411.2875).

Heteronmin (**3**): ^13^C NMR (CDCl_3_, 100 MHz) data: δ 171.3 (C, OAc), 170.1 (C, OAc), 135.3 (C, C-17), 114.4 (CH, C-24), 101.6 (CH, C-25), 80.5 (CH, C-12), 69.3 (CH, C-16), 64.1 (CH, C-18), 58.7 (CH, C-9), 56.5 (CH, C-5), 54.6 (CH, C-14), 42.7 (C, C-13), 42.0 (CH_2_, C-3), 41.8 (CH_2_, C-7), 39.9 (CH_2_, C-1), 38.0 (C, C-10), 37.4 (C, C-8), 33.2 (CH_3_, C-19), 33.2 (C, C-4), 28.0 (CH_2_, C-15), 27.2 (CH_2_, C-11), 21.3 (CH_3_, OAc), 21.2 (CH_3_, OAc), 21.0 (CH_3_, C-20), 18.6 (CH_2_, C-6), 18.1 (CH_2_, C-2), 17.3 (CH_3_, C-21), 16.3 (CH_3_, C-22), 8.7 (CH_3_, C-23). Selective ^1^H NMR (CDCl_3_, 400 MHz) data: δ 6.76 (1H, s, H-25), 6.16 (1H, s, H-24), 5.35 (1H, m, H-16), 3.42 (1H, d, *J* = 11.6 Hz, H-12), 2.43 (1H, s, H-18), 0.91 (3H, s, H_3_-21), 0.84 (6H, s, H_3_-19 and H_3_-22), 0.82 (3H, s, H-20). 

Scalarafuran (**8**): ^13^C NMR (CDCl_3_, 125 MHz) data: δ 171.2 (C, OAc), 139.0 (CH, C-24), 137.3 (CH, C-25), 134.5 (C, C-18), 120.9 (C, C-17), 79.6 (CH, C-12), 68.1 (CH, C-16), 58.6 (CH, C-9), 56.6 (CH, C-5), 54.0 (CH, C-14), 42.0 (CH_2_, C-3), 41.6 (CH_2_, C-7), 40.1 (C, C-13), 39.8 (CH_2_, C-1), 37.4 (C, C-10), 37.4 (C, C-8), 33.3 (CH_3_, C-19), 33.2 (C, C-4), 27.8 (CH_2_, C-11), 24.6 (CH_2_, C-15), 21.3 (CH_3_, OAc), 21.3 (CH_3_, C-20), 18.8 (CH_3_, C-23),18.6 (CH_2_, C-6), 18.1 (CH_2_, C-2), 17.4 (CH_3_, C-21), 16.2 (CH_3_, C-22), Selective ^1^H NMR (CDCl_3_, 500 MHz) data: δ 7.53 (1H, d, *J* = 1.5 Hz, H-25), 7.26 (1H, s, H-24), 5.76 (1H, dd, *J* = 8.5, 8.0 Hz, H-16), 3.60 (1H, d, *J* = 10.5 Hz, H-12), 1.26 (3H, s, H_3_-23), 0.91 (3H, s, H_3_-21), 0.85 (3H, s, H_3_-22), 0.84 (3H, s, H_3_-19), 0.81 (3H, s, H_3_-20). 

### 3.4. Cytotoxicity Testing

Cell lines were purchased from the American Type Culture Collection (ATCC). Cytotoxicity assays of compounds **1**–**8** were performed using the MTT [3-(4,5-dimethylthiazol-2-yl)-2,5-diphenyltetrazolium bromide] colorimetric method [11,12]. 

### 3.5. Molecular Mechanics Calculations

Implementation of the MM2 force filed in Chem3D Pro software [13] was used to calculate the molecular models.

## 4. Conclusions

Previous chemical investigations of sponges of the genus *Hippospongia* have led to the isolation and identification of various metabolites [14,15,16,17,18,19,20,21,22,23,24,25,26,27,28,29,30,31,32,33,34,35,36]. Some of these have been found to possess several kinds of biological activities, such as isocitrate lyase (ICL) inhibitory [14], RCE protease inhibitory [15] and cytotoxic [16,17,18,19,20,21] activities. In the present study, two new sesterterpenoids, hippospongides A and B (**1** and **2**), together with six known scalarane sesterterpenoids were isolated from the sponge *Hippospongia* sp*.* Compounds **3**–**5** showed significant cytoxicities against DLD-1, HCT-116, T-47D and K562 cell lines. However, the new compounds **1** and **2** and the other known compounds had no significant activity. Furthermore, it is worth mentioning that these compounds are the first pentacyclic sesterterpene and scalarane-type sesterterpenes to be reported from this genus. However, this genus is often confused with *Hyattella* and the sesterterpenoids are not likely to assist in chemical differentiation of the species.

## Supplementary Files

Supplementary File 1: PDF-Document (PDF, 3039 KB)
