# Supplementary material for: Cytotoxic Sesterterpenoids from a Sponge Hippospongia sp
Source: Mar Drugs. 2012 Apr 27;10(5):987–97. doi: 10.3390/md10050987 (PMC3397461; doi:10.3390/md10050987)
Supplement: Supplementary File 1: — PDF-Document (PDF, 3039 KB) [file marinedrugs-10-00987-s001.pdf]

Supporting Information

Cytotoxic Sesterterpenoids from the Sponge *Hippospongia* sp.

Yu-Chia Chang, Shang-Wei Tseng, Li-Lian Liu, Yalan Chou, Yuan-Shing Ho, Mei-Chin Lu and Jui-Hsin Su*

Table of Contents

S1. Table of Contents

S2. ^{1}H NMR spectrum of **1** in $CDCl_3$ at 500 MHz.

S3. ^{13}C NMR spectrum of **1** in $CDCl_3$ at 125 MHz.

S4. ^{1}H NMR spectrum of **2** in $CDCl_3$ at 500 MHz.

S5. ^{13}C NMR spectrum of **2** in $CDCl_3$ at 125 MHz.

S6. ^{1}H NMR spectrum of **3** in $CDCl_3$ at 400 MHz.

S7. ^{13}C NMR spectrum of **3** in $CDCl_3$ at 100 MHz.

S8. ^{1}H NMR spectrum of **4** in $CDCl_3$ at 400 MHz.

S9. ^{13}C NMR spectrum of **4** in $CDCl_3$ at 100 MHz.

S10. ^{1}H NMR spectrum of **5** in $CDCl_3$ at 400 MHz.

S11. ^{13}C NMR spectrum of **5** in $CDCl_3$ at 100 MHz.

S12. ^{1}H NMR spectrum of **6** in $CDCl_3$ at 500 MHz.

S13. ^{13}C NMR spectrum of **6** in $CDCl_3$ at 125 MHz.

S14. ^{1}H NMR spectrum of **7** in $CDCl_3$ at 400 MHz.

S15. ^{13}C NMR spectrum of **7** in $CDCl_3$ at 100 MHz.

S16. ^{1}H NMR spectrum of **8** in $CDCl_3$ at 500 MHz.

S17. ^{13}C NMR spectrum of **8** in $CDCl_3$ at 125 MHz.

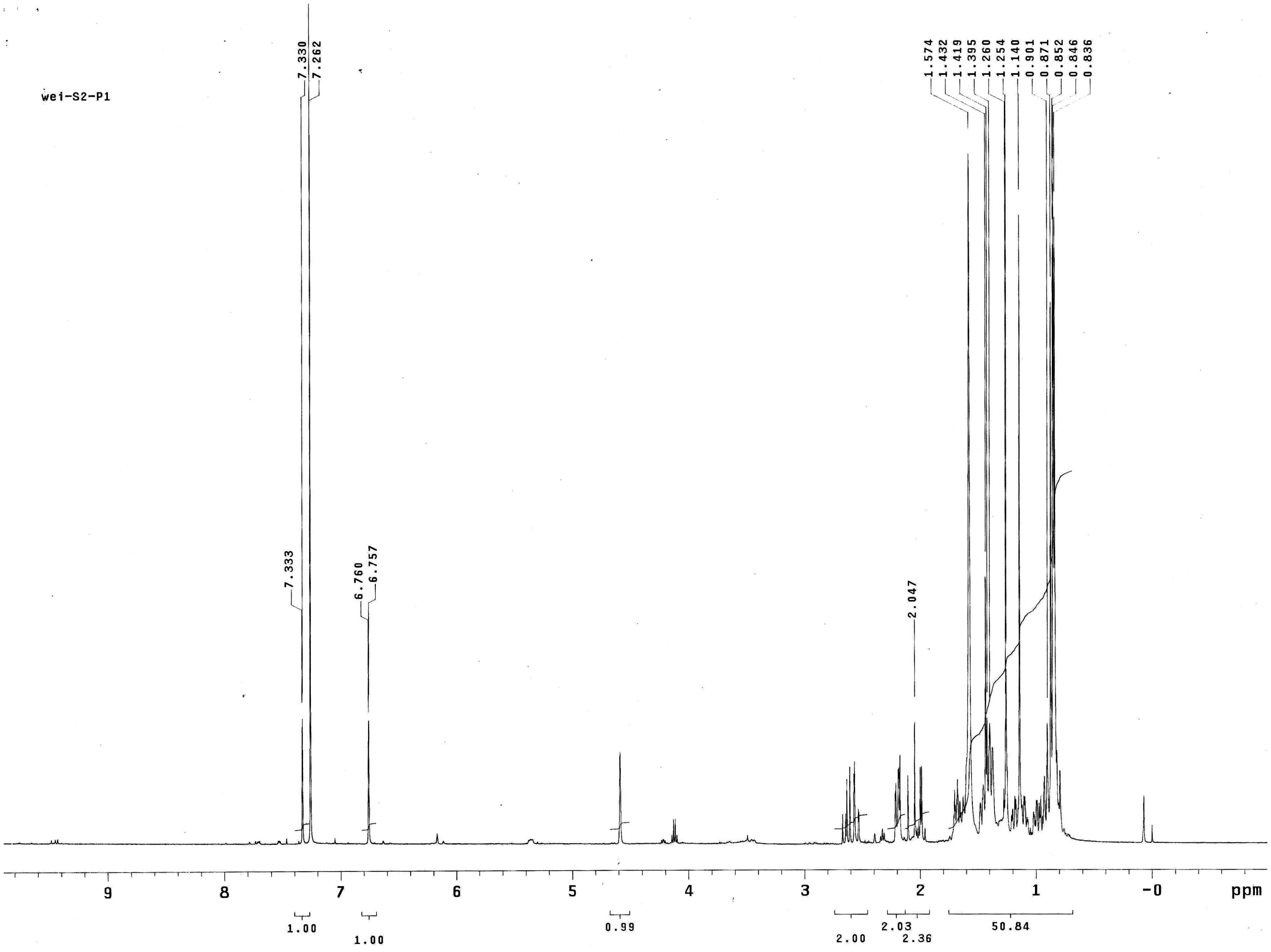

S2. ^{1}H NMR spectrum of **1** in $CDCl_3$ at 500 MHz.

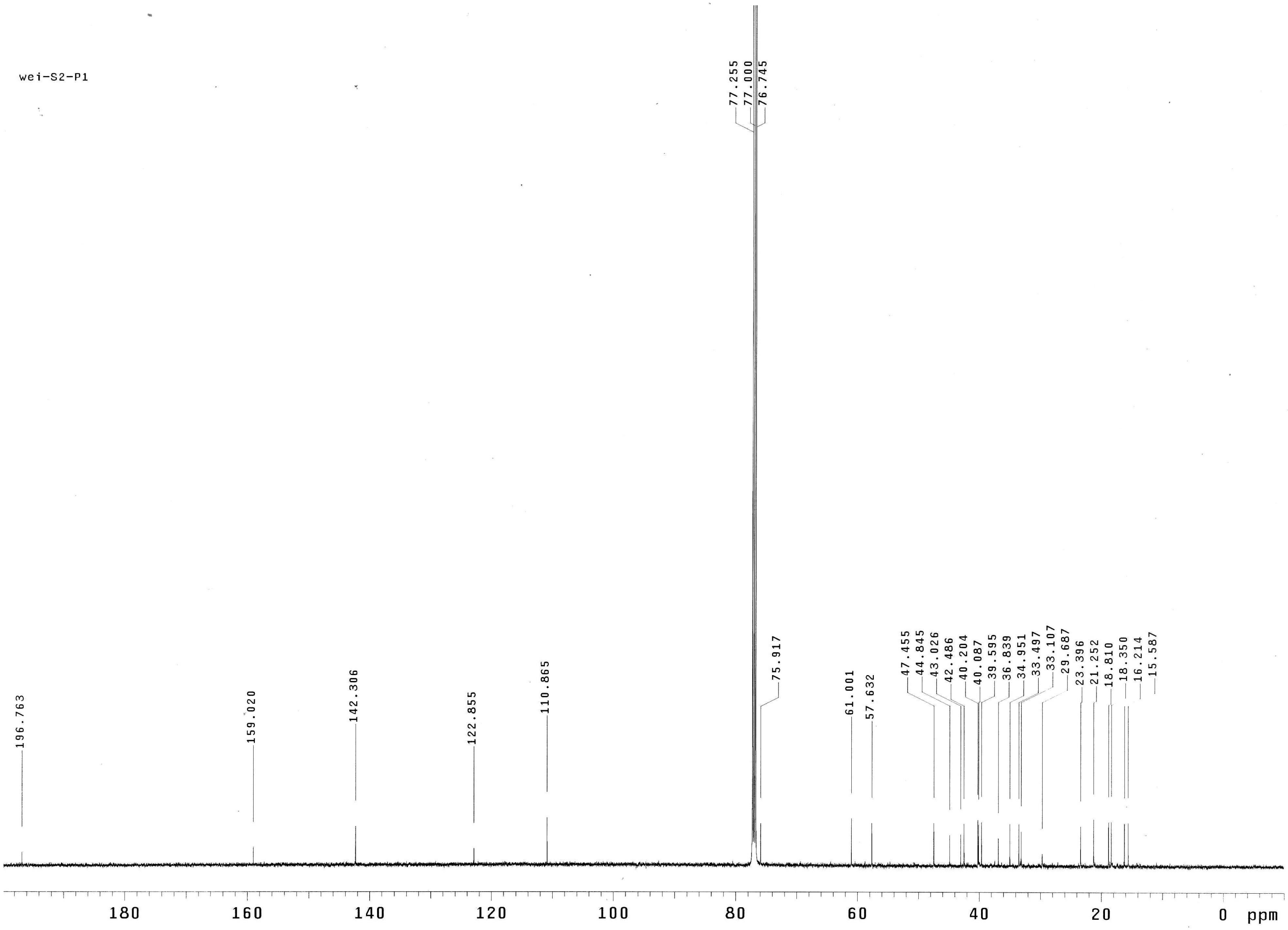

S3. ^{13}C NMR spectrum of **1** in $CDCl_3$ at 125 MHz.

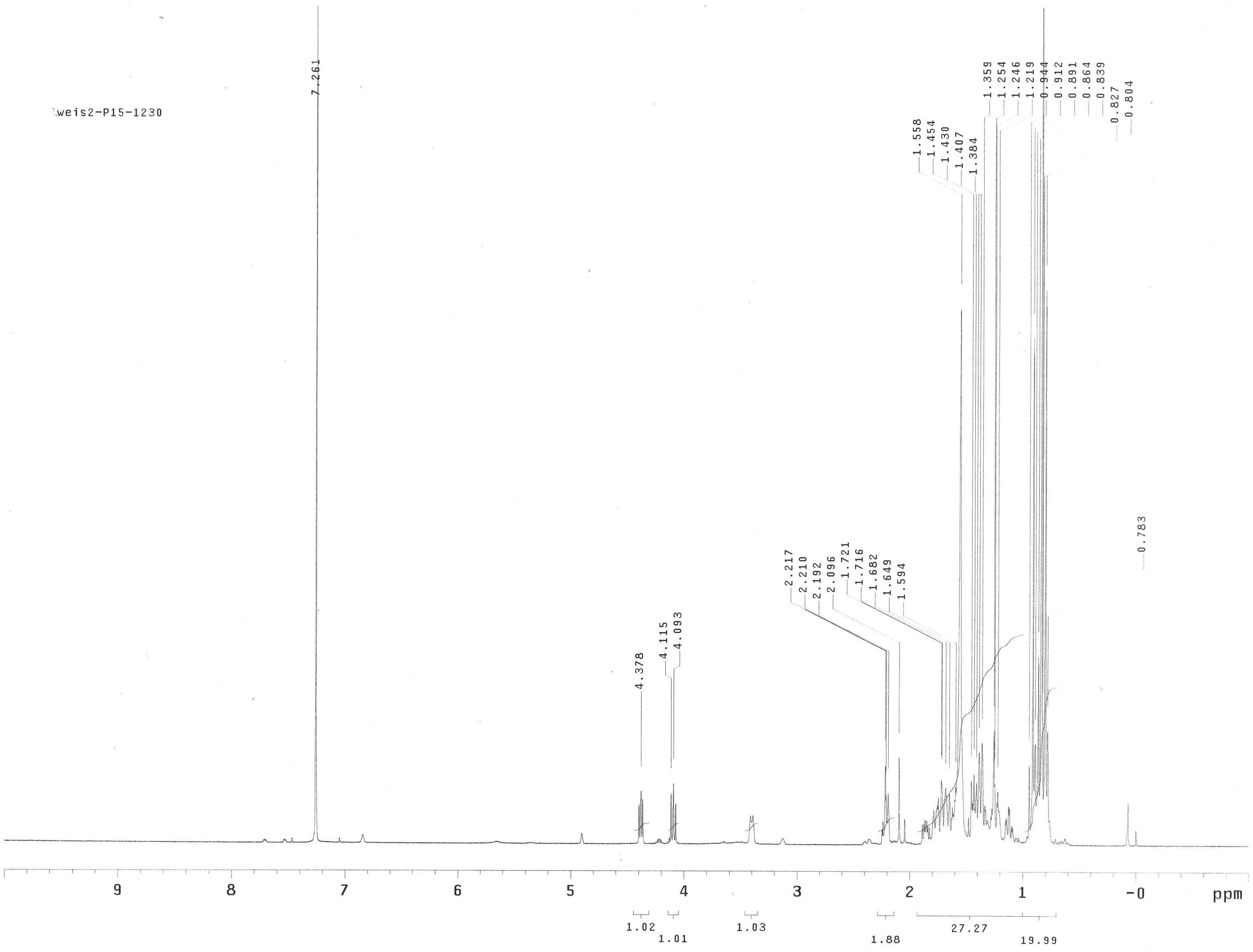

S4. ^{1}H NMR spectrum of **2** in $CDCl_3$ at 500 MHz.

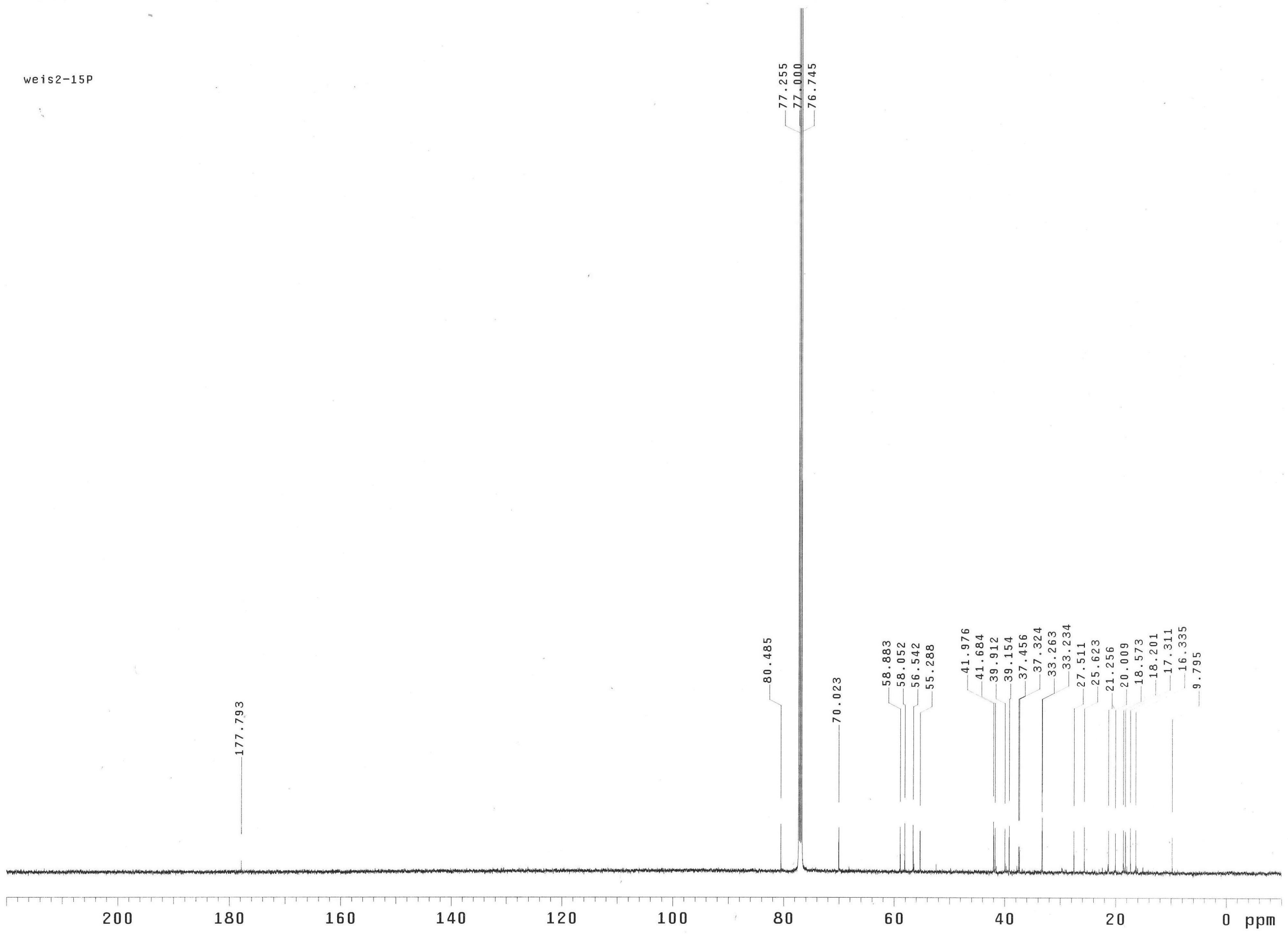

S5. ^{13}C NMR spectrum of **2** in $CDCl_3$ at 125 MHz.

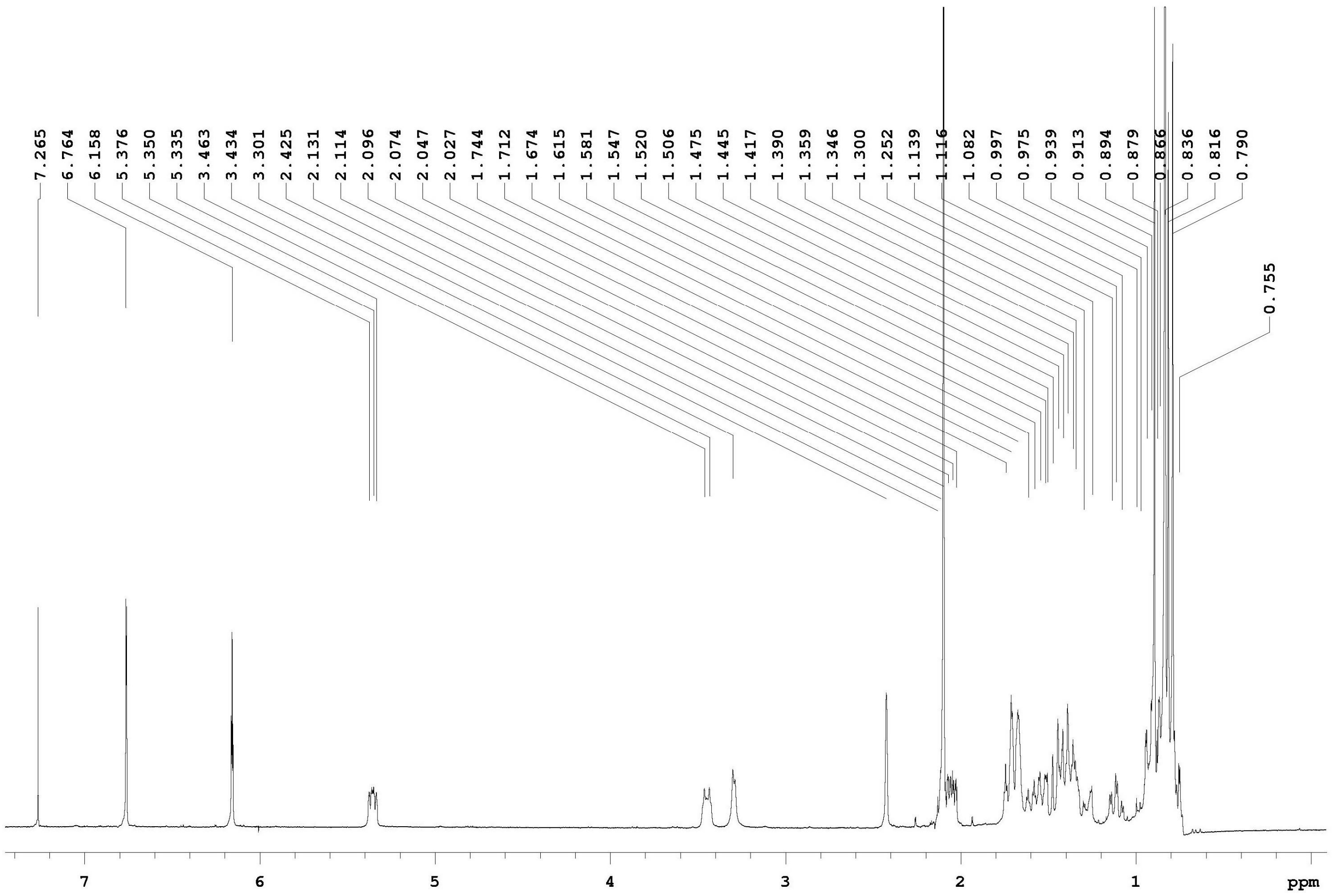

S6. ^{1}H NMR spectrum of **3** in $CDCl_3$ at 400 MHz.

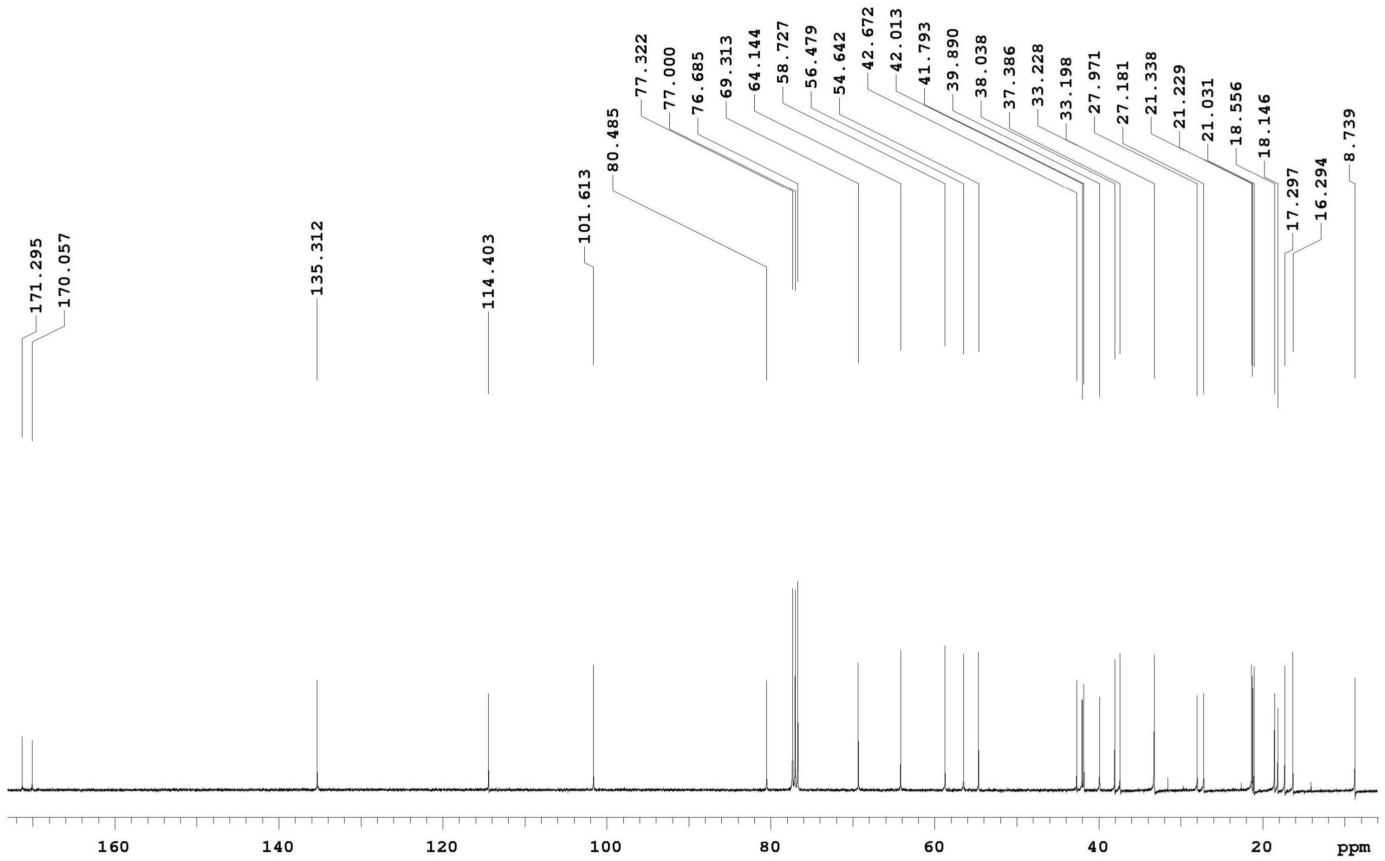

S7. ^{13}C NMR spectrum of **3** in $CDCl_3$ at 100 MHz.

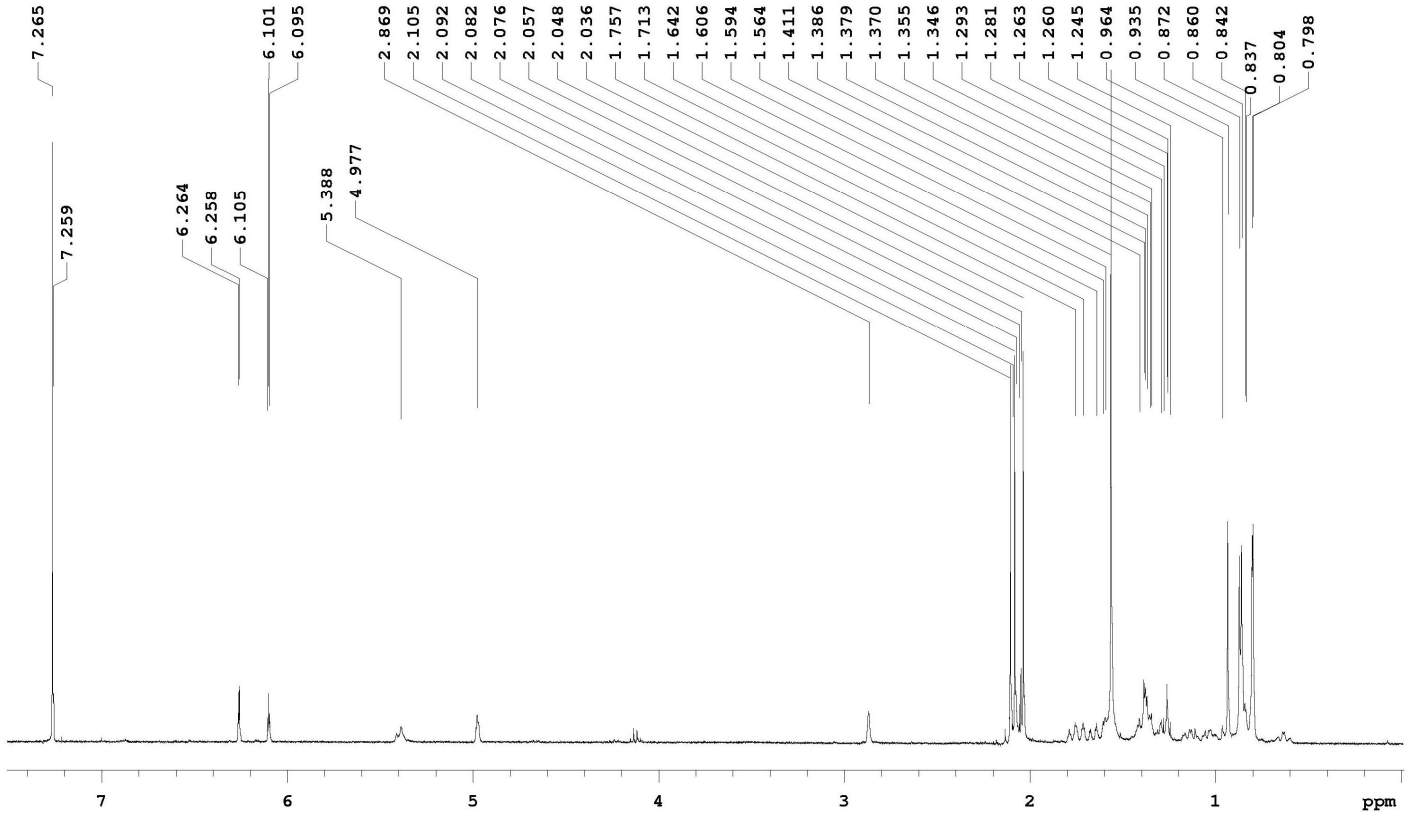

S8. ^{1}H NMR spectrum of **4** in $CDCl_3$ at 400 MHz.

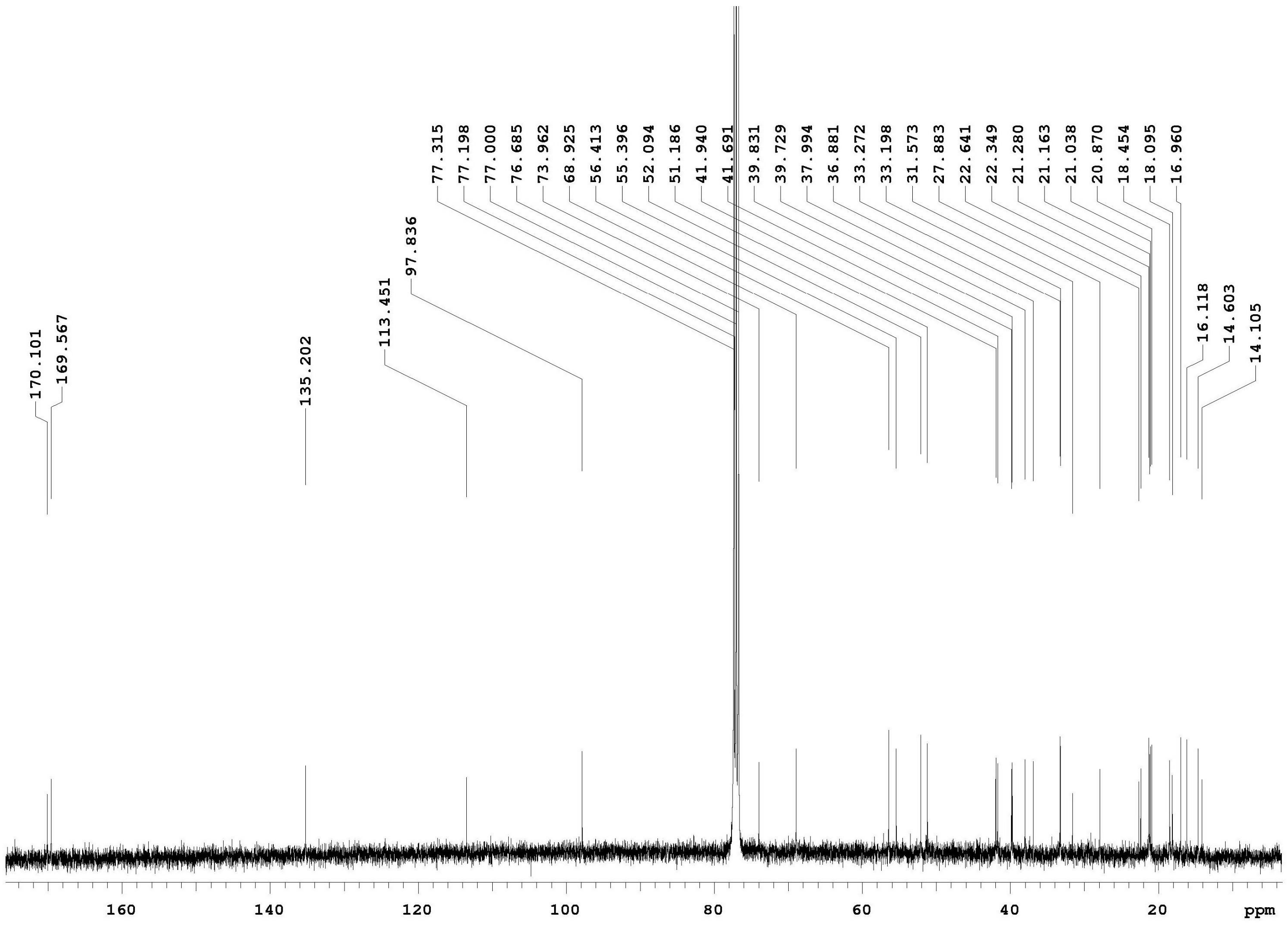

S9. ^{13}C NMR spectrum of **4** in $CDCl_3$ at 100 MHz.

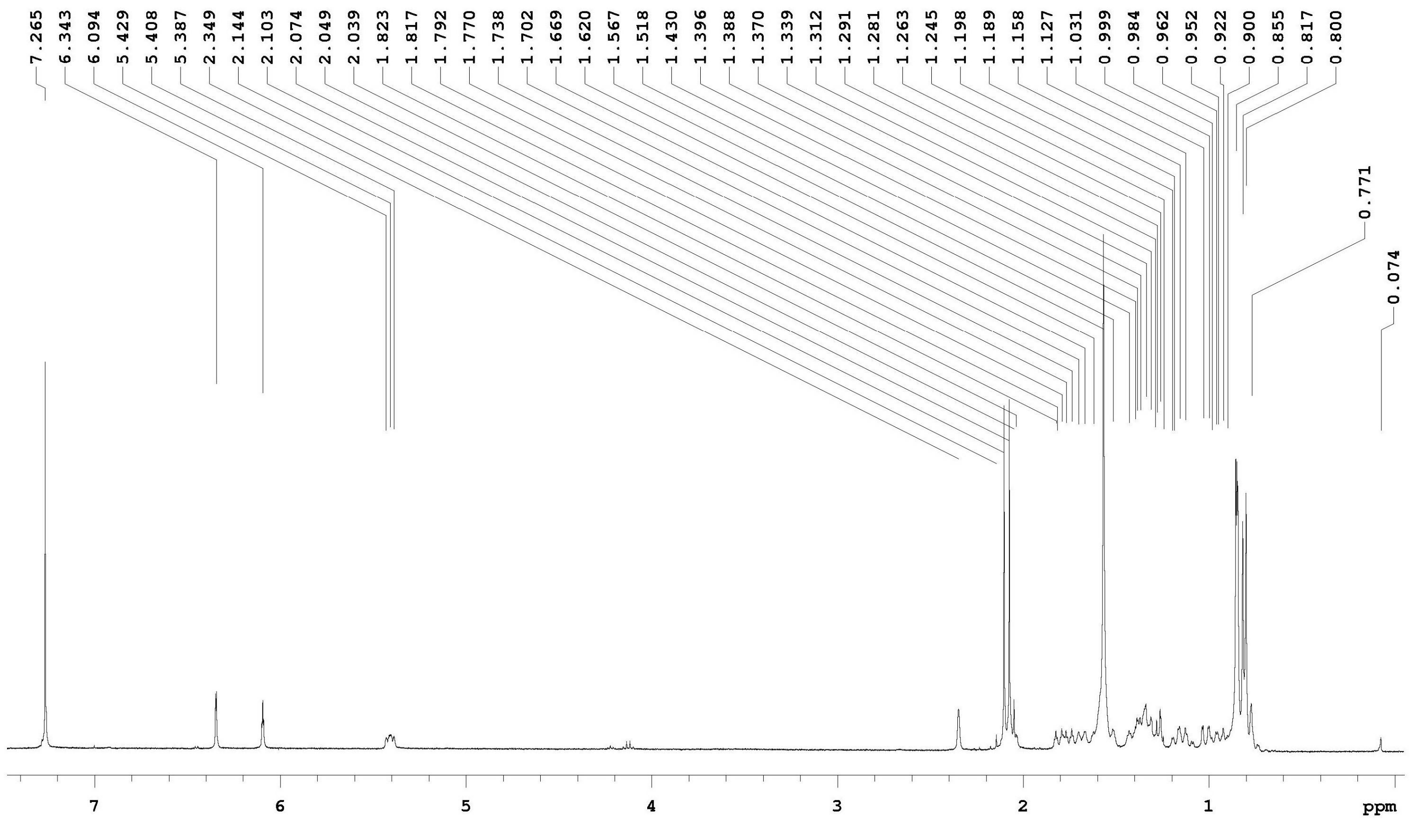

S10. ^{1}H NMR spectrum of **5** in $CDCl_3$ at 400 MHz.

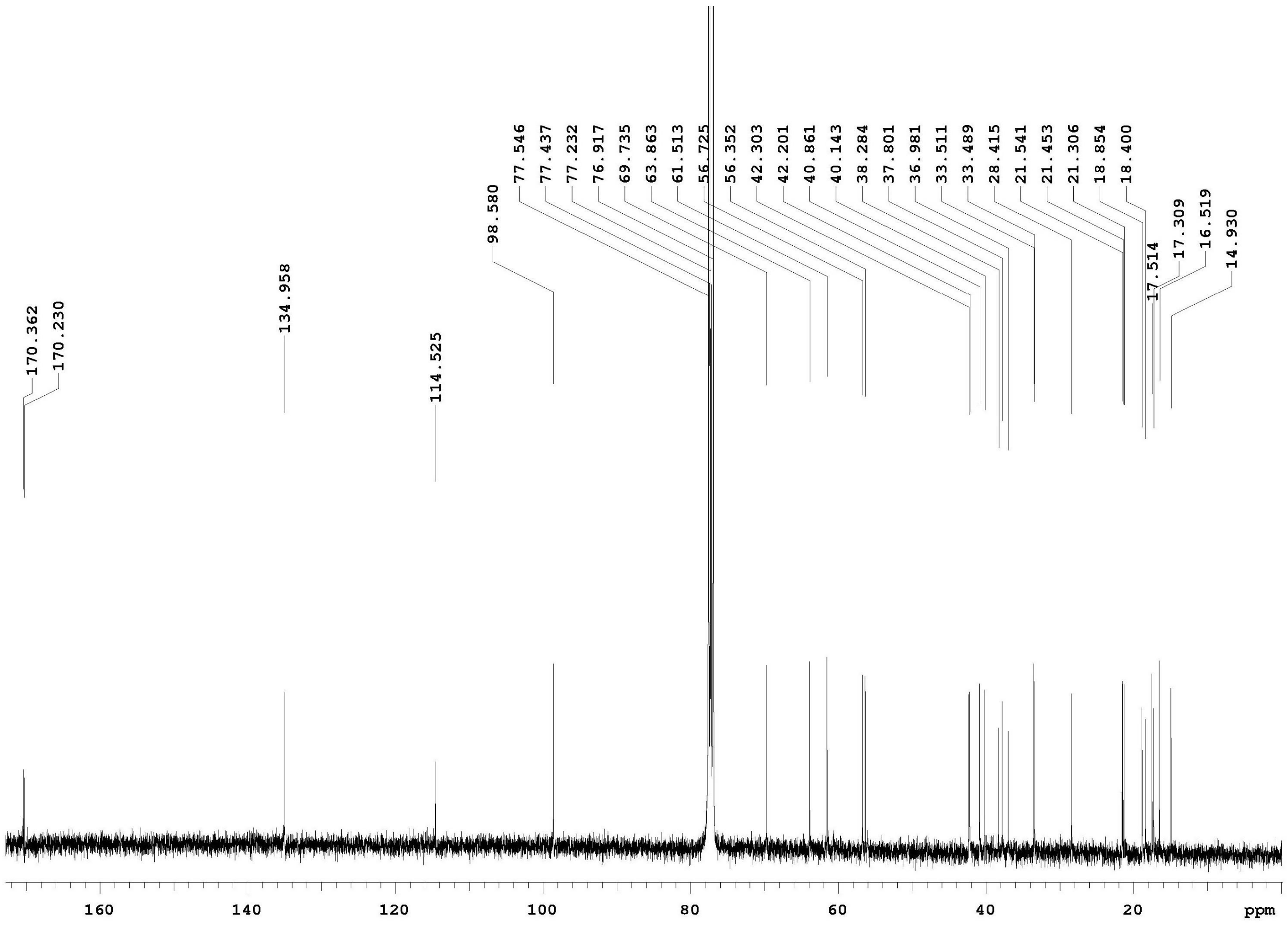

S11. ^{13}C NMR spectrum of **5** in $CDCl_3$ at 100 MHz.

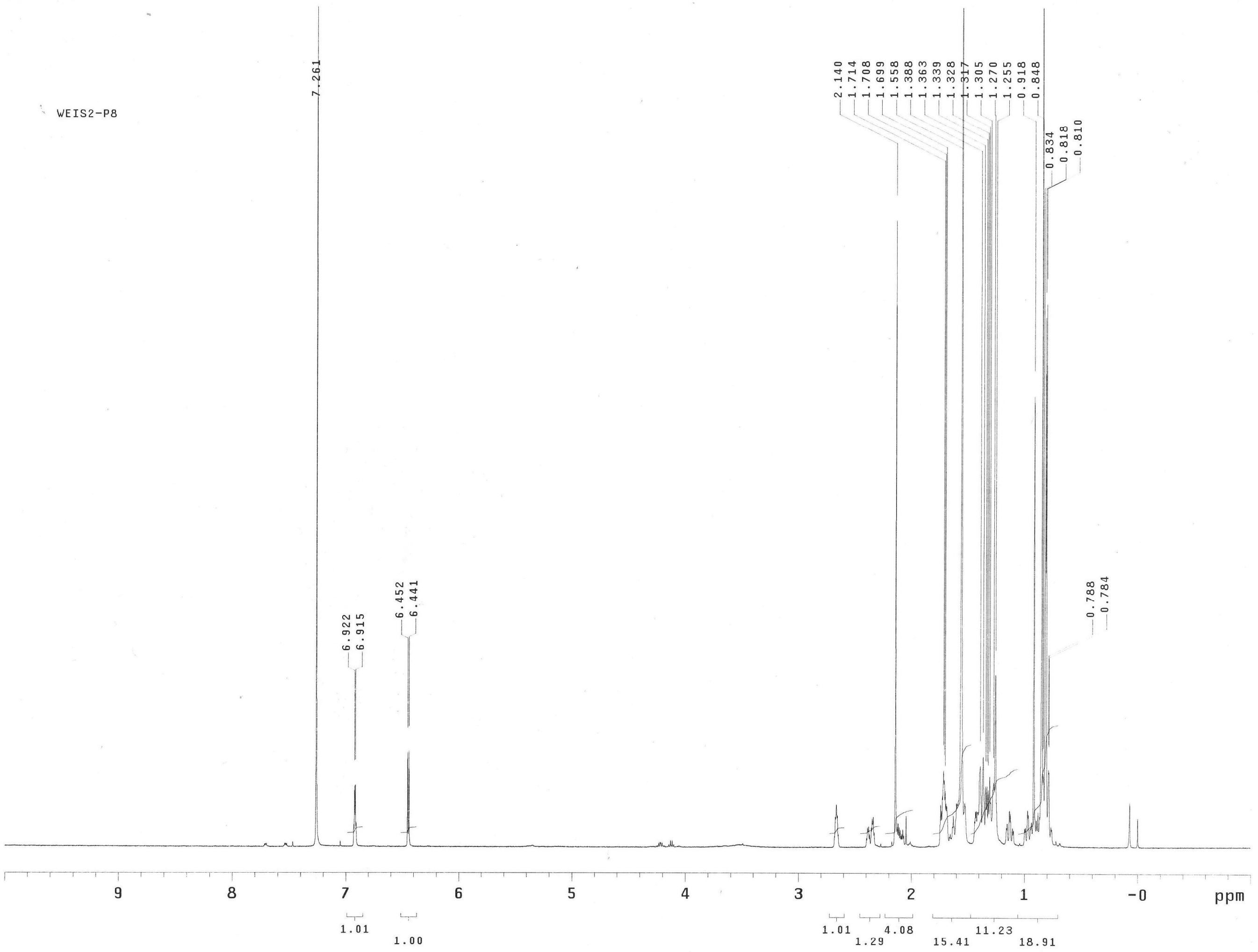

S12. ^{1}H NMR spectrum of **6** in $CDCl_3$ at 500 MHz.

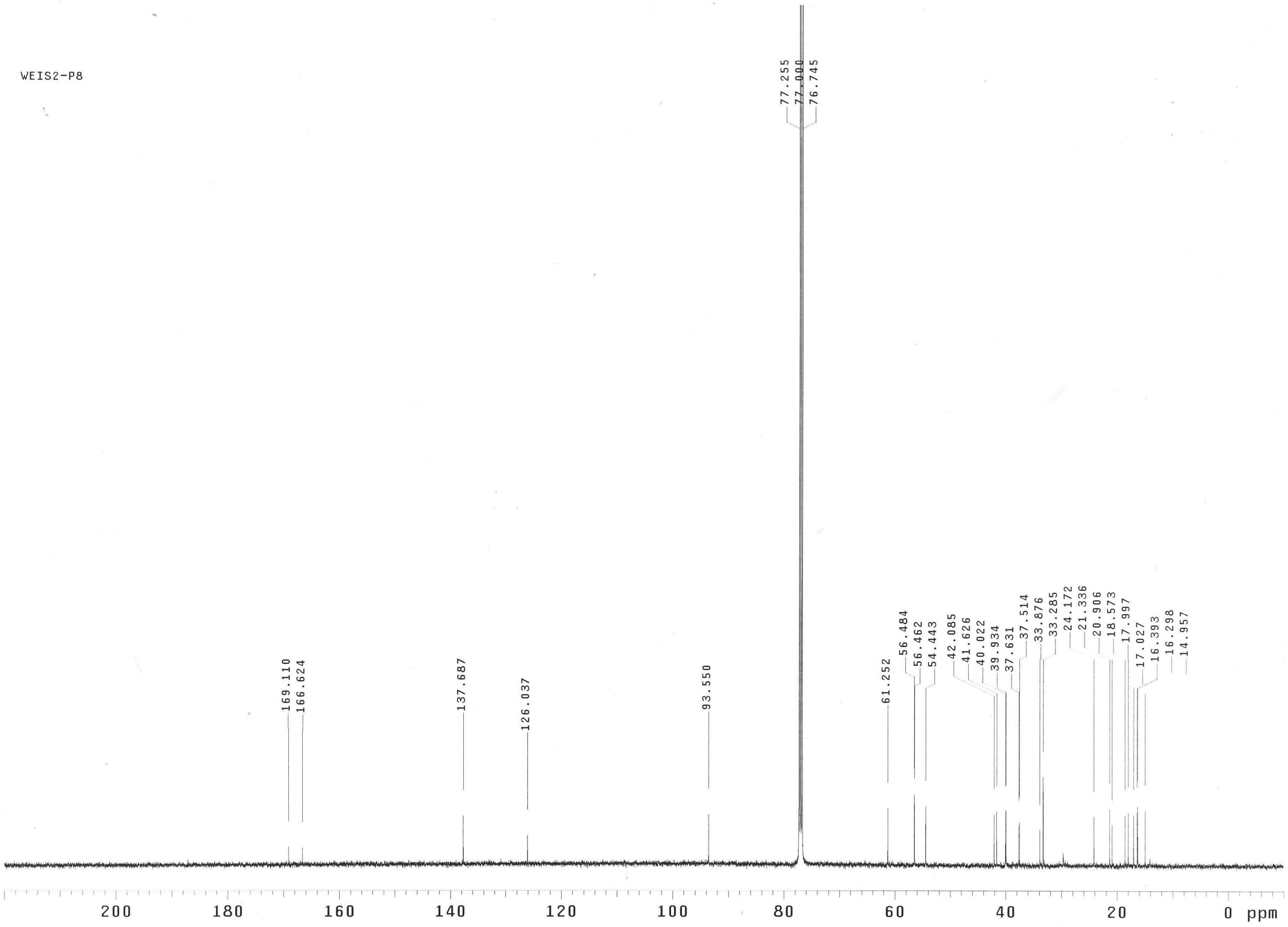

S13. ^{13}C NMR spectrum of **6** in $CDCl_3$ at 125 MHz.

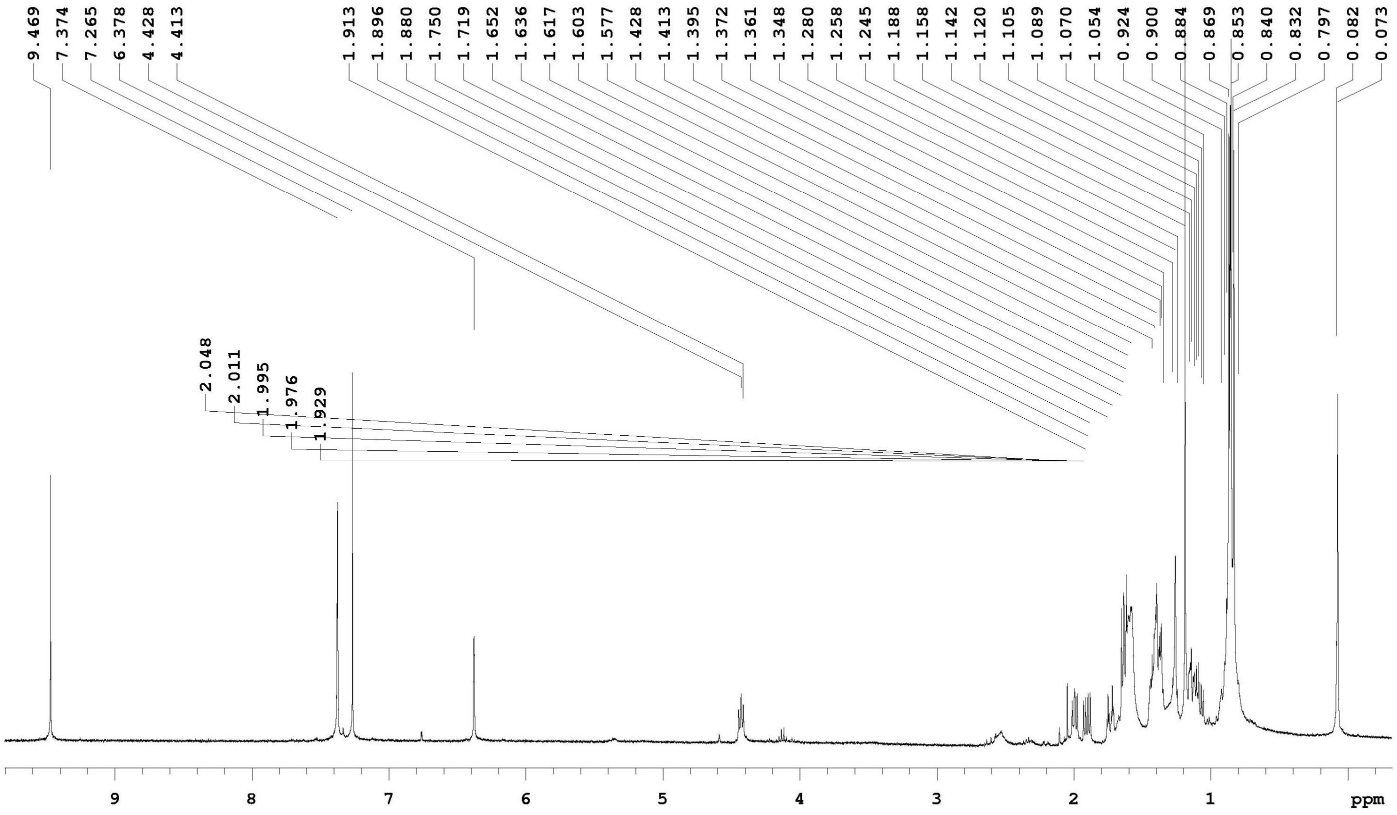

S14. ^{1}H NMR spectrum of **7** in $CDCl_3$ at 400 MHz.

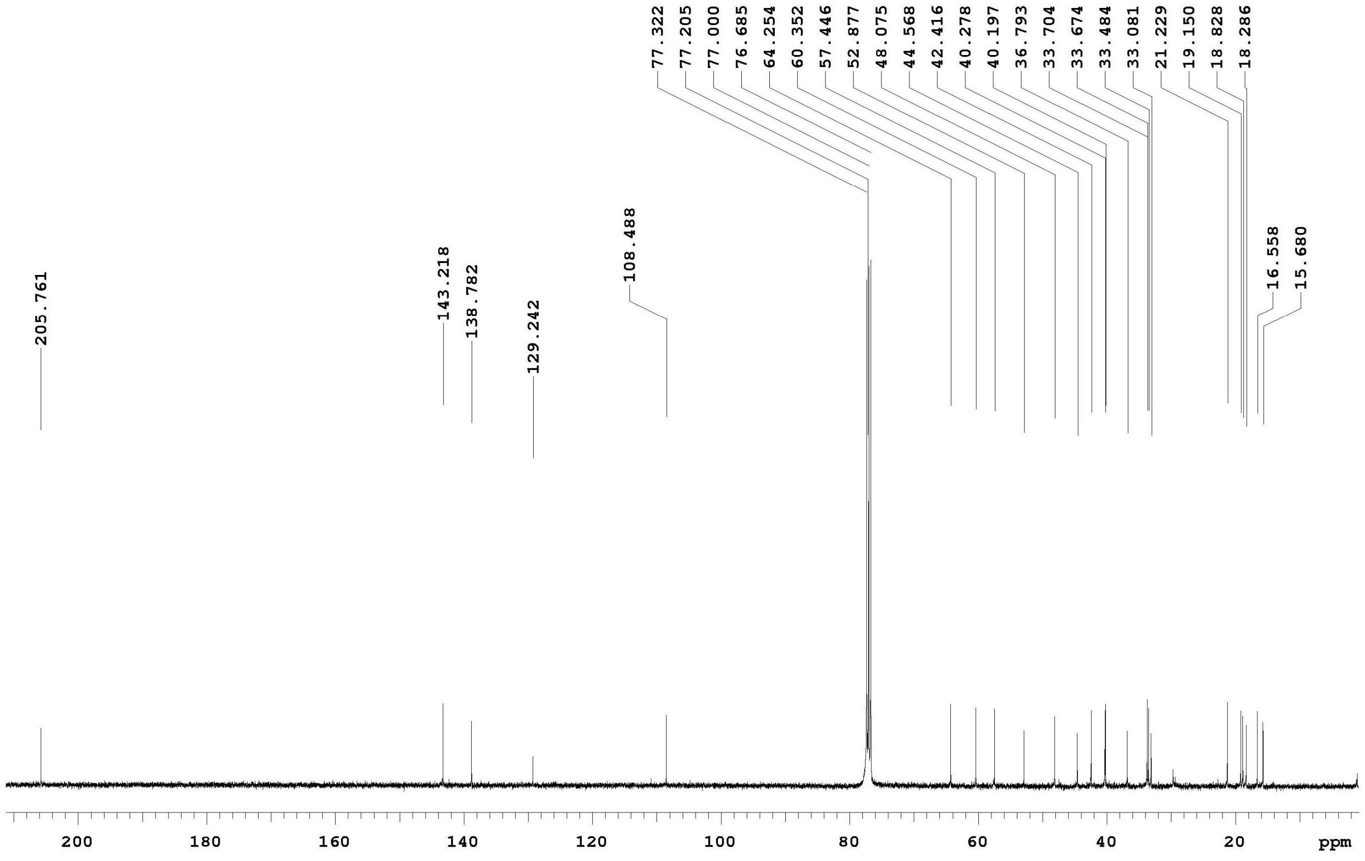

S15. ^{13}C NMR spectrum of **7** in $CDCl_3$ at 100 MHz.

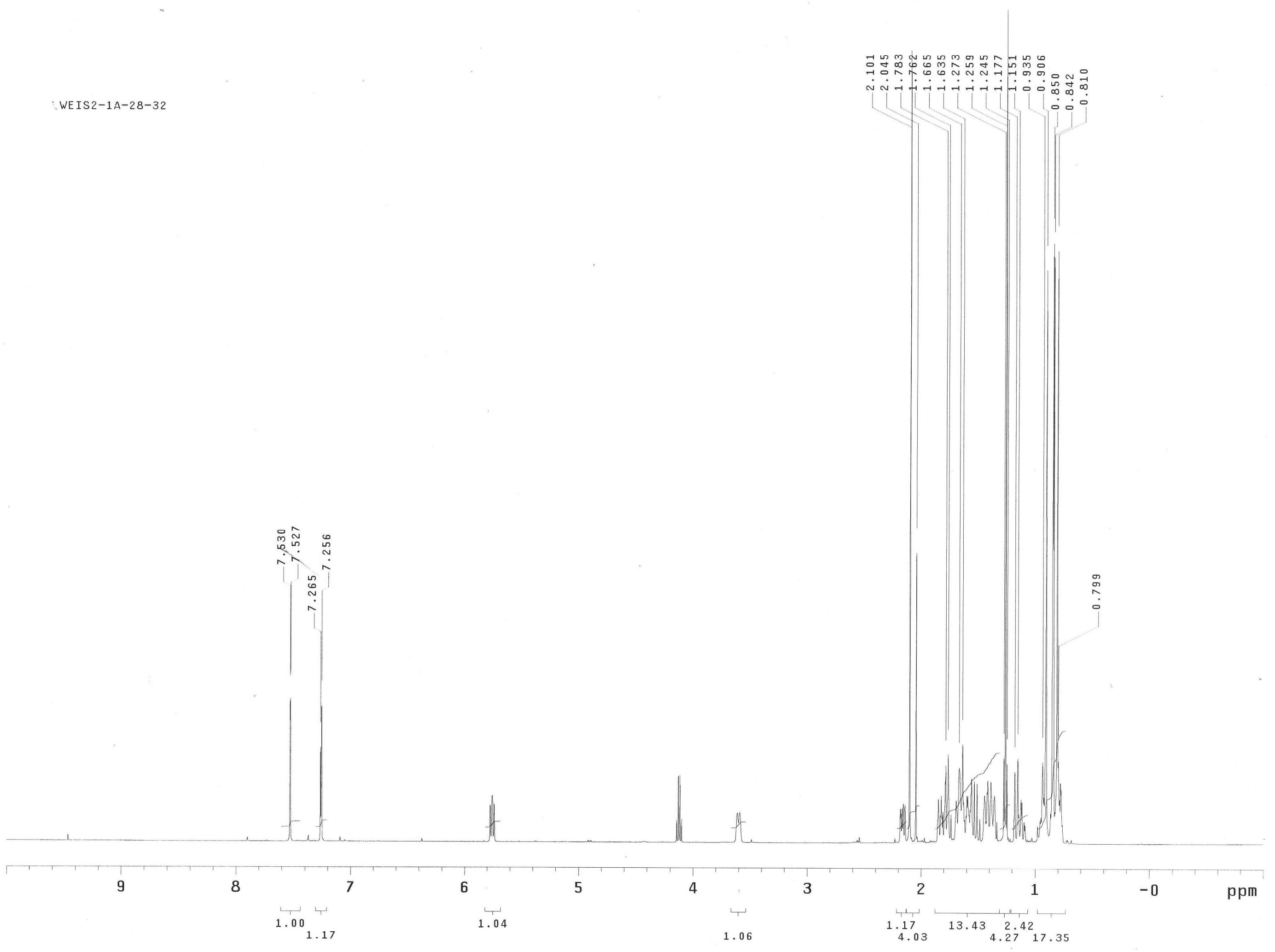

S16. ^{1}H NMR spectrum of **8** in $CDCl_3$ at 500 MHz.

WEIS2-1A-28-32

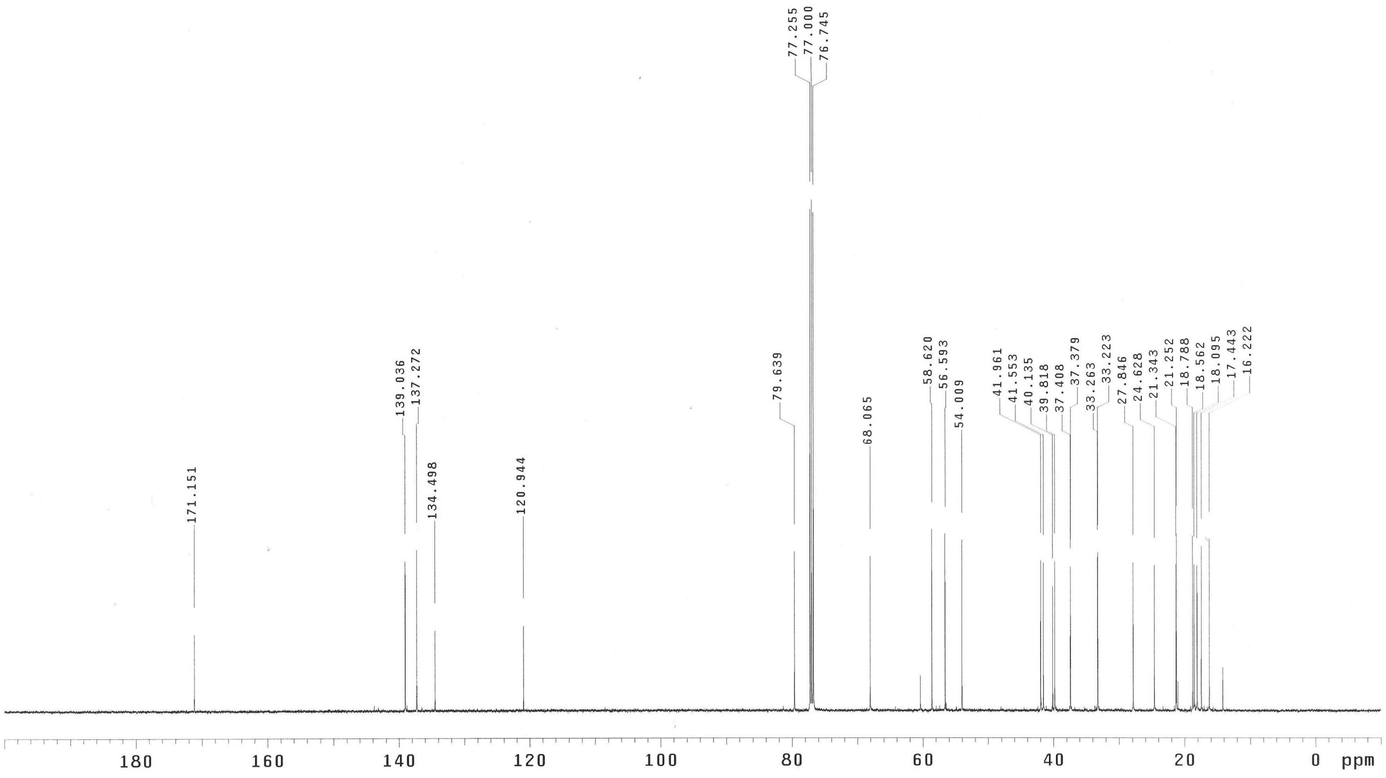

S17. ^{13}C NMR spectrum of **8** in $CDCl_3$ at 125 MHz.
